# Burnout among Nurses during Coronavirus Disease 2019 Outbreak in Shiraz

**DOI:** 10.31661/gmj.v9i0.1956

**Published:** 2020-12-26

**Authors:** Mahsa Kamali, Ahmad Kalateh Sadati, Mohammad Reza Khademi, Sulmaz Ghahramani, Leila Zarei, Seyede Zahra Ghaemi, Reza Tabrizi, Maryam Akbari, Nasrin Shokrpour, Arash Mani, Seyed Taghi Heydari, Kamran Bagheri Lankarani

**Affiliations:** ^1^Health Policy Research Center, Institute of Health, Shiraz University of Medical Sciences, Shiraz, Fars, Iran; ^2^Department of Social Sciences, Yazd University, Yazd, Iran; ^3^English Department, Shiraz University of Medical Sciences, Shiraz, Iran; ^4^Research Center for Psychiatry and Behavioral Sciences, Shiraz University of Medical Sciences, Shiraz, Iran

**Keywords:** COVID-19, Nursing Burnout, Emotional Exhaustion

## Abstract

**Background:**

The function of healthcare workers, particularly nursing staff, in taking care of coronavirus disease 2019 (COVID-19) patients, cannot be overemphasized. As the pandemic lasts, burnout among the nursing staff needs to be considered as an important challenge. This was aimed to assess the nurses’ burnout and factors affecting this variable.

**Materials and Methods:**

In this cross-sectional study, Maslach Burnout Inventory was completed by 261 nurses in Shiraz hospitals (Iran) in April 2020. This questionnaire addresses different aspects, including emotional exhaustion, personal achievement, and depersonalization, to determine the intensity of perceived burnout among nurses during the outbreak.

**Results:**

Our data demonstrated that the nurses’ burnout in Shiraz hospitals during the COVID-19 pandemic was high (64.6%). Emotional exhaustion and depersonalization were observed in 63.6 and 53.3 percent of the participants, respectively. Moreover, the rate of successful personal achievement among these nurses was >97%. Work experience <10 years (P=0.016), hospital ward (P=0.044), the number of deaths observed by nurses during the COVID-19 pandemic (P<0.001), and the total number of shifts during the COVID-19 pandemic (P=0.006) had a positive correlation with emotional exhaustion.

**Conclusion:**

Workload and stress resulting from the COVID-19 outbreak seem to be one of the major causes of emotional exhaustion in nurses. The emotional exhaustion among nurses must be considered in epidemics, such as COVID-19.

## Introduction

Coronavirus disease 2019 (COVID-19) began in Wuhan, China, and spread rapidly worldwide [1,2]. Severe pneumonia and mechanical ventilation requiring acute respiratory distress syndrome may occur just the same as some shock and multi-failures [3]. Despite the acute complications of this disease in some patients, there is still no approved treatment for COVID-19, and some drugs are under investigation [4,5]. Hospitals play a critical role in dealing with this epidemic, and as most of the offered care is supportive, health care providers are one of the primary sources to control this pandemic. In this regard, the nursing staff plays a pivotal role. At the same time, they are at an increased risk of burnout.

Freudenberg introduced the term ‘burnout’ in 1974 when he observed the loss of motivation among volunteers at a mental health clinic [6]. This phenomenon has different dimensions [7] and encompasses three unique aspects; inefficacy, exhaustion, and cynicism [8]. Emotional exhaustion, sense of reduced personal accomplishment, and depersonalization are attributed to burnout [9]. Maslach proposed the hypothesis that burnout occurs when an individual is in long-term contact with at least one of the items such as workload, power, compensation, culture, justice, and values [8]. Nowadays, the Maslach questionnaire is the most common tool to assess burnout [10]. Job burnout can affect all aspects of a person’s daily life, such as psychological health, personal satisfaction, wish to leave a job, reduced performance and effectiveness, and even aggressive behaviors [11]. This occurrence usually happens due to a variety of societal impacts [12]. Numerous research has demonstrated that burnout, particularly in nursing workers, can affect the professional standard of care and treatment and the health of patients, and thus arouses work dissatisfaction and high staff turnover [7,9,13,14]. As the nurse staffs are usually the first-line the health care frontline, their burnout rate has been reported to be higher, compared to other health care workers [15]. During the current pandemic, this rate might even be higher. There are many studies on nursing burnout. For example, Poncet *et al*. (2007) illustrated that the burnout rate among critical care nurses was 33% [16]. Our previous study (2016) assessed the rate of nursing burnout in public and private hospitals in Shiraz, Iran. The results of this study showed that the nurses in the internal wards of public hospitals had a constant burnout rate [7]. Nurses play a critical and vital role in COVID-19 epidemics; as such, it is of paramount importance to address their job issues. Recent studies on nursing problems have also made attempts to deal with such issues [17]. Nurses should use protective equipment for long hours to take care of patients who experience severe forms of the disease [17]. Several studies have evaluated the experiences of nurses in recent outbreaks and mostly addressed anxiety and anger [18]. A study conducted in South Korea during the outbreak of COVID-19 on burnout revealed the mediating role of the work, emotional, and financial factors in this regard [19]. They also reported a significant relationship between burnout and emotion regulation and no significant effect of burnout on economic issues [20]. Another qualitative study showed burnout in nurses when they had so many patients under their observation who were positive in this virus outbreak [20]. The first cases of COVID-19 deaths were reported in Iran in February 2020 [21]. According to the most recent policy of the Ministry of Health in Iran, all hospitals are supposed to admit patients suspected of COVID-19 infection. The nursing departments of the hospitals need a plan to control their space, staff, and equipment to be prepared for patients who are given optimal treatments. The high number of patients, unknown nature of the disease, and fear of transmission have raised numerous side-effects among nurses, including burnout. The impact of burnout on nurses during the COVID-19 pandemic in Iran has not been thoroughly detected, and a few research studies have examined this issue. Hence, the present research aimed to examine the COVID-19 pandemic with regard to the burnout experienced by nurses at hospitals in Shiraz, Iran.

## Materials and Methods

This cross-sectional study included 261 nurses who work at hospitals affiliated with Shiraz University of Medical Sciences from March 20 to April 3, 2020. The nurses had regular shifts during the outbreak and were working in different wards, including wards devoted to COVID-19 patients, Emergency room (E.R.), internal medicine, surgery, intensive care unit (ICU), and coronary care unit (CCU) ward during the last six months. According to Rezaei *et al*. [22], the Iranian nurses’ burnout rate was measured at 36%, α=0.05, and a precision rate of 0.072. In this regard, the sample size was also estimated to be 171 persons; however, 261 nurses were included in this study for more precision. The collected data included demographic features, job characteristics (including years of work experience, caring for dying COVID-19 patients, the total number of shifts since the start of the COVID-19 outbreak in Shiraz [March 2020]), and Maslach Burnout Inventory scores [7].

A valid Persian version of the Maslach Burnout Inventory was used in this study, which encompassed nine items on emotional exhaustion, eight items on depersonalization, and five items on personal accomplishment [23]. The Maslach Burnout inventory has frequency and intensity ratings. Because of redundancy, the intensity rating was used in this study [24]. The Persian version of this questionnaire was evaluated and validated by Moalemi *et al*. as they reported Cronbach’s alpha values for this questionnaire and its three dimensions >0.7 [25]. In this study, a nurse with a high score for either depersonalization and/or emotional exhaustion dimensions was considered to have at least one of the manifestations of job burnout [24]. The level of emotional exhaustion was classified to be high (scores >26), medium (scores=17-26), and low (scores <17). Regarding the depersonalization dimension, the total scores of >12, 7–12, and <7 were considered as high, medium, and low depersonalization, respectively. Regarding the personal achievement dimension, the high, medium, and low achievements were represented by scores >39, 32–38, and <32, respectively [23]. A trained nurse went to different wards of the hospital, explained the objectives of the study, and invited the hospital nurses to cooperate and complete the questionnaire. After receiving informed consent, the questionnaires were completed for each nurse by trained interviewers. The research was conducted with regard to the ethical guidelines of the Statement of Helsinki and the American Sociology Association [26]. This research was authorized by the Ethics Committee of Shiraz University of Medical Sciences (IR.SUMS.REC.1398.877). The research was also granted by Research Deputy of Shiraz University of Medical Sciences (Code: 98-01-01-22074).

###  Statistical Analysis

 The data were analyzed using SPSS software version 18 (SPSS Inc., Chicago, IL, USA). The mean and standard deviation were considered to report descriptive statistics. Chi-square test was also used to compare the means of emotional exhaustion and depersonalization by age, gender, marital status, hospital ward, and other qualitative variables. P<0.05 was considered to be statistically meaningful.

## Results

 A total of 261 nurses participated in this study, included 176 (67.2%) females and 85 (32.4%) males. The mean age of the nurses was 28.91±6.87 years. Regarding the participants’ marital status, there were 143 (54.6%) single and 94 (35.9%) married, and 3(1.1%) divorced individuals. Regarding different wards of the hospital, most nurses were working in the internal wards (n=98, 37.4%). Furthermore, most nurses’ experience belonged to senior nurses (n=208, 79.4%). The descriptive statistics revealed that the means of emotional exhaustion, depersonalization, and personal accomplishment were 29.22±9.64, 7.41±5.04, and 18.53±6.19, respectively. The mean score of burnout was 55.17±15.38. Moreover, 166 (63.6%), 68 (26.1%), and 27 (10.3%) participants had high, medium, and low levels of emotional exhaustion, respectively. Furthermore, 39 (14.9%), 83 (31.8%), and 139 (53.3%) of the participants had high, medium, and low levels of depersonalization, respectively. Additionally, 0 (0.0%), 7 (2.7%), and 254 (97.3%) nurses experienced low, medium, and high levels of personal accomplishment, respectively. On the other hand, the prevalence of burnout in this research was 64.6%. As shown in Table-1, emotional exhaustion was not significant in the number of nurses’ shifts in the wards devoted to COVID-19 patients (P=0.071). The age (P=0.023), marital status (P=0.046), work experience <10 years (P=0.016), hospital ward (all the nurses were working in the hospital wards) (P=0.044), the number of deaths observed by nurses during the COVID-19 pandemic (P<0.001), and the total number of shifts since the start of the COVID-19 outbreak in Shiraz (P=0.006) were correlated with emotional exhaustion. The depersonalization dimension was not correlated with age, marital status, number of shift work in the COVID-19 specific ward, and the total number of shifts since the start of the COVID-19 outbreak in Shiraz. On the other hand, work experience (P=0.036), hospital ward (P=0.009), and the number of deaths observed by nurses during the COVID-19 pandemic (P=0.026) were correlated with depersonalization.

## Discussion

During the past couple of years, there has been increased attention to a policy on how the features of the work organization affect different nursing outcomes. Numerous studies have investigated the association between work organization’s variables and job dissatisfaction. The remarkable role of nursing forces during the COVID-19 pandemic prompted us to study burnout among the nurses. Our findings showed that the prevalence of burnout was 64.6%. In other words, a majority of the nurses in this study suffered from burnout. Moreover, 63.6% of the nurses were suffering from different levels of emotional exhaustion. In general, the results of this study are consistent with those of other studies on COVID-19 [17]. The estimated incidence of burnout among 43000 nurses from the United States, Canada, Britain, Scotland, and New Zealand varied from 32%(Scotland) to 54%(United States) [27]. The prevalence of burnout in Iran also varied from 22 % to 51% during the study period in the wards, where the nurses were working [28,29]. In a study conducted in 2018 to assess the burnout level in nurses after the Health Transformation Program (HSEP) in Iran (2014), the findings revealed that nurses’ level of burnout significantly changed from 52.00 ± 21.0 to 51.0 ±19.7 [30]. It should be noted that these studies evaluated burnout during the period when all countries were not engaged with COVID-19 pandemic even though high levels of burnout observed among the nurses in this study (64.6%) might be a red flag for the healthcare system. The health system needs to adopt proper measures to fight against this issue as soon as possible. In this study, 63.6% of the nurses stated high levels of emotional exhaustion. A meta-analysis revealed a medium level of emotional exhaustion among Iranian nurses (36%) [22].

Moreover, its total prevalence among hospital nurses was similar to that reported in another country. For example, the prevalence of high levels of emotional exhaustion among nurses was 36% and 36.2% in Canada and England, respectively [31]. According to these findings, it may be argued that the COVID-19 pandemic has significantly enhanced burnout resulting from chronic stress, incredibly emotional exhaustion, among nurses at Shiraz hospitals. The lack of adequate infrastructures and the dramatic daily increases of new cases have a direct detrimental effect on the overall standard of treatment and pose pressure-related symptoms in nurses [20,32]. The deficiency in human resource management (lower nurses/ patient ratio) is a universal and critical issue faced by the health care systems during the COVID-19 pandemic; therefore, nurses are faced with an increased risk of physical and psychological pressure, arousing exhaustion among them [20,32]. Furthermore, the findings demonstrated a significant association between the total number of shifts during the COVID-19 pandemic and emotional exhaustion. It could be attributed to accumulated work overload [33]. Excessive workload could be due to increased working hours and/or inadequate nurses to patient ratio. The findings from this analysis describe the second form of workload arising from the COVID-19 pandemic [32]. The highest levels of emotional exhaustion and depersonalization among nurses aged below 25 years were reported in the current research. Senior nurses (aged≥ 35 years) had the lowest emotional exhaustion percentage. This research showed the relationship between emotional exhaustion and work experience with depersonalization [34]. Highly experienced nurses would probably have further accomplishments. They would also accomplish much of their objectives; however, they may suffer from higher levels of burnout as well [13,35]. In addition, our study findings revealed that young nurses became emotionally exhausted during the COVID-19 pandemic. Our study demonstrated a strong association between the number of deaths observed by nurses during the COVID-19 pandemic and emotional exhaustion. Moreover, nurses working in surgical wards significantly exhibited more emotional exhaustion. It might be due to the stressful situations they faced as the further case by case investigation is recommended [35,36]. Further quantitative and qualitative studies on nursing stress during this outbreak are also recommended. Several studies have demonstrated a significant association between burnout and overwork dimensions among nurses [37]. It is found that the feeling of work pressure is correlated with psychiatric disorders [38]. The COVID-19 pandemic has raised concerns about mental health, especially among health workers, including nurses. Mental disabilities are one of the leading causes of impairment at work. This thus leads to the reduction of potential power among health workers, including nurses, as such, they fail to perform their jobs efficiently.

## Limitations

 One of the limitations of the study was the lack of access to the wards of different hospitals due to the policies adopted to prevent the spread of the coronavirus. In this regard, it was difficult to compare the concerned variables in COVID-19 ward’s nurses and the same variables in the nurses working in other wards.

## Conclusion

 The findings of this research indicate that nursing burnout has been rising in Shiraz hospitals during the COVID-19 pandemic. A statistically significant increase in emotional exhaustion was observed in this study. It seems that stress and job pressures are strongly correlated with this increase; hence, the policymakers need to adopt measures to alleviate these symptoms and improve treatment standards.

## Conflict of Interest

 The authors declare that they have no competing interests.

**Table 1:** Association between Demographic Variables and Job Characteristics with the Prevalence of Emotional Exhaustion and Depersonalization

**Variables** **Low**	**Emotional Exhaustion** **n( %)**			**P-value** **Low**	**Depersonalization** **n( %)**			**P-value**
	**Moderate**	**High**			**Moderate**	**High**		
**Age (year)**								
<25	5(4.5)	35(31.3)	72(64.3)	0.023	56(50)	38(33.9)	18(16.1)	0.116
25-34	12(12)	24(24)	64(64)		51(51)	37(37)	12(12)	
≥35	10(20.4)	9(18.4)	30(61.2)		32(65.3)	8(16.3)	9(18.4)	
**Marital status**								
Single or Divorced	10(6.8)	45(30.8)	91(62.3)	0.046	78(53.4)	51(34.9)	17(11.6)	0.227
Married	14(14.9)	19(20.2)	61(64.9)		52(55.3)	25(26.6)	17(18.1)	
**Hospital ward**								
Covid-19	5(12.5)	9(22.5)	26(65.0)	0.044	22(55.0)	9(22.5)	9(22.5)	0.009
Emergency room	8(10.8)	27(36.5)	39(52.7)		40(54.1)	24(32.4)	10(13.5)	
Internal	6(6.1)	25(25.5)	67(68.4)		48(49.0)	42(42.9)	8(8.2)	
Surgical	2(8.0)	3(12.0)	20(80.0)		12(48.0)	7(28.0)	6(24.0)	
ICU & CCU	6(25.0)	4(16.7)	14(58.3)			17(70.8)	1(4.2)		6(25.0)
**Work experience**								
≤1	5(8.2)	18(29.5)	38(62.3)	0.016	37(60.7)	16(26.2)	8(13.1)	0.036
1-5	6(4.9)	35(28.50)	82(66.7)		56(45.5)	51(41.5)	16(13.0)	
5-10	5(20.0)	7(28.0)	13(52.0)		13(52.0)	8(32.0)	4(16.0)	
>10	11(21.2)	8(15.4)	33(63.5)		33(63.5)	8(15.4)	11(21.2)	
Number of witnessed deaths*								
0	16(25.8)	13(21.0)	33(53.2)	<0.001	39(62.9)	12(19.4)	11(17.7)	0.026
1-5	7(5.9)	29(24.4)	83(69.7)		52(43.7)	47(39.5)	20(16.8)	
˃5	4(5.0)	26(32.5)	50(62.5)		48(60.0)	24(30.0)	8(10.0)	
**Number of shift work in COVID-19 ward**								
<5	5(20.8)	5(20.8)	14(58.3)	0.071	17(70.8)	4(16.7)	3(12.5)	0.107
6-10	7(18.9)	8(21.6)	22(59.5)		25(67.6)	7(18.9)	5(13.5)	
11-20	8(14.3)	15(26.8)	33(58.9)		30(53.6)	21(37.5)	5(8.9)	
21-30	1(1.4)	23(32.4)	47(66.2)		37(52.1)	23(32.4)	11(15.5)	
>30	6(8.2)	17(23.3)	50(68.5)		30(41.1)	28(38.4)	15(20.5)	
**Total number of shifts during the COVID-19 pandemic**								
<5	9(15.3)	12(20.3)	38(64.4)	0.006	34(57.6)	11(18.6)	14(23.7)	0.056
6-10	8(18.6)	6(14.0)	29(67.4)		22(51.2)	13(30.2)	8(18.6)	
11-20	8(17.8)	14(31.1)	23(51.1)		26(57.8)	17(37.8)	2(4.4)	
21-30	0(0.0)	15(32.6)	31(67.4)		27(58.7)	13(28.3)	6(13.0)	
>30	2(2.9)	21(30.9)	45(66.2)		30(44.1)	29(42.6)	9(13.2)	

* The number of patients who died during the COVID-19 pandemic; nurses have seen. **Results for high scores in either depersonalization and/or emotional exhaustion dimensions are considered as having at least one manifestation of professional burnout. This finding was 166 (63.6%) when only high emotional exhaustion was considered, and 166 (64.4%) when either depersonalization and/or emotional exhaustion dimensions were evaluated.
